# Virtual Pharmacist: A Platform for Pharmacogenomics

**DOI:** 10.1371/journal.pone.0141105

**Published:** 2015-10-23

**Authors:** Ronghai Cheng, Ross Ka-Kit Leung, Yao Chen, Yidan Pan, Yin Tong, Zhoufang Li, Luwen Ning, Xuefeng B. Ling, Jiankui He

**Affiliations:** 1 Department of Biology, South University of Science and Technology of China, Shenzhen, China; 2 Division of Genomics and Bioinformatics, The Chinese University of Hong Kong, Hong Kong, China; 3 Departments of Surgery, Stanford University, Stanford, California, United States of America; University of Southern California, UNITED STATES

## Abstract

We present Virtual Pharmacist, a web-based platform that takes common types of high-throughput data, namely microarray SNP genotyping data, FASTQ and Variant Call Format (VCF) files as inputs, and reports potential drug responses in terms of efficacy, dosage and toxicity at one glance. Batch submission facilitates multivariate analysis or data mining of targeted groups. Individual analysis consists of a report that is readily comprehensible to patients and practioners who have basic knowledge in pharmacology, a table that summarizes variants and potential affected drug response according to the US Food and Drug Administration pharmacogenomic biomarker labeled drug list and PharmGKB, and visualization of a gene-drug-target network. Group analysis provides the distribution of the variants and potential affected drug response of a target group, a sample-gene variant count table, and a sample-drug count table. Our analysis of genomes from the 1000 Genome Project underlines the potentially differential drug responses among different human populations. Even within the same population, the findings from Watson’s genome highlight the importance of personalized medicine. Virtual Pharmacist can be accessed freely at http://www.sustc-genome.org.cn/vp or installed as a local web server. The codes and documentation are available at the GitHub repository (https://github.com/VirtualPharmacist/vp). Administrators can download the source codes to customize access settings for further development.

## Introduction

Since the first release of the human genome in 2000, there has been continuing interest to understand genetic variants among individuals. The Single Nucleotide Polymorphism Database (dbSNP) is a collection of such variations [1]. Genetic variations can affect drug responses involving efficacy and safety to different extents, and the outcomes also affect drug development, prescription, and patient care [2]. For examples, the effective dosage of the drug warfarin is strongly affected by genetic variants of the P450 cytochrome CYP2C9 and the vitamin K epoxide reductase complex VKORC1 [3]. The labels for warfarin and other drugs, such as abacavir, clopidogrel, prasugrel, and irinotecan have already incorporated pharmacogenetic information [4]. To meet the need for high-quality genotypic and phenotypic information, the National Institute of Health initiated the Pharmacogenetics Research Network [5], which led to the development of the Pharmacogenetics and Pharmacogenomics Knowledge Base (PharmGKB), a curated resource that contains the relationships between drugs, diseases/phenotypes, and genes involved in pharmacokinetics and pharmacodynamics [6]. In 2010, the US Food and Drug Administration (FDA) issued a black-box warning of diminished clopidogrel effectiveness in poor metabolizers and suggested testing for the CYP2C19 genotype.

Low-throughput methods including Polymerase Chain Reaction (PCR) are common options for detecting drug-related gene variants, because of the low technology requirements and operation cost. In recent years, however, the cost of high-throughput sequencing has dramatically reduced and the “$1000 genome” [7–9] may be realized in the near future, when single nucleotide polymorphism (SNP) genotyping chips will be replaced by whole-genome sequencing [10]. With the generation of more and more data, their interpretation can become the bottleneck [11]. wANNOVAR [12] was developed to annotate genetic variants with disease associations. Similarly, Karczewski et al. [13] developed a platform called Interpretome that can be used to estimate risk for diseases. 23andMe is a service company that provides genetic testing for inherited disorders and ancestry-related analysis [14]. Other related work includes integration of multiple databases for annotation [15], visualization or manipulation [16], and analysis and knowledge discovery [15, 17].

Tools need to be developed for interpreting high-throughput data of personal genomes and for identifying the variations that affect drug response [8, 12, 18–21]. Here, we present Virtual Pharmacist (VP), a secure online platform that can be used to interpret the potential impact of individual genetic variations on drug response, based on the high-quality resources from PharmGKB [6], dbSNP [1], and The DrugBank database [22], which is a comprehensive resource that curates knowledge about drugs and their targets.

## Methods

VP has a modular design to accommodate enhancement features such as implementation of prediction algorithms and/or incorporation of additional analysis functionalities. VP uses technologies based on open standards, such as Hypertext Preprocessor (PHP) and Python for backend processing. JavaScript and Cascading Style Sheets (CSS) were used to construct a user-friendly Graphical User Interface. MySQL was chosen as the core database management system for fast and flexible data retrieval.

### Data security

We developed a three-fold security strategy to protect user data privacy and security. First, VP generates a folder named with a random string to store user data; second, the folder and files are deleted automatically 7 days after uploading; and third, we adopted open source software development and deposited the whole package with detailed documentation at GitHub. Administrators can download the source codes and customize access settings for their organizations and users at any level.

### Workflow

**Fig 1:**
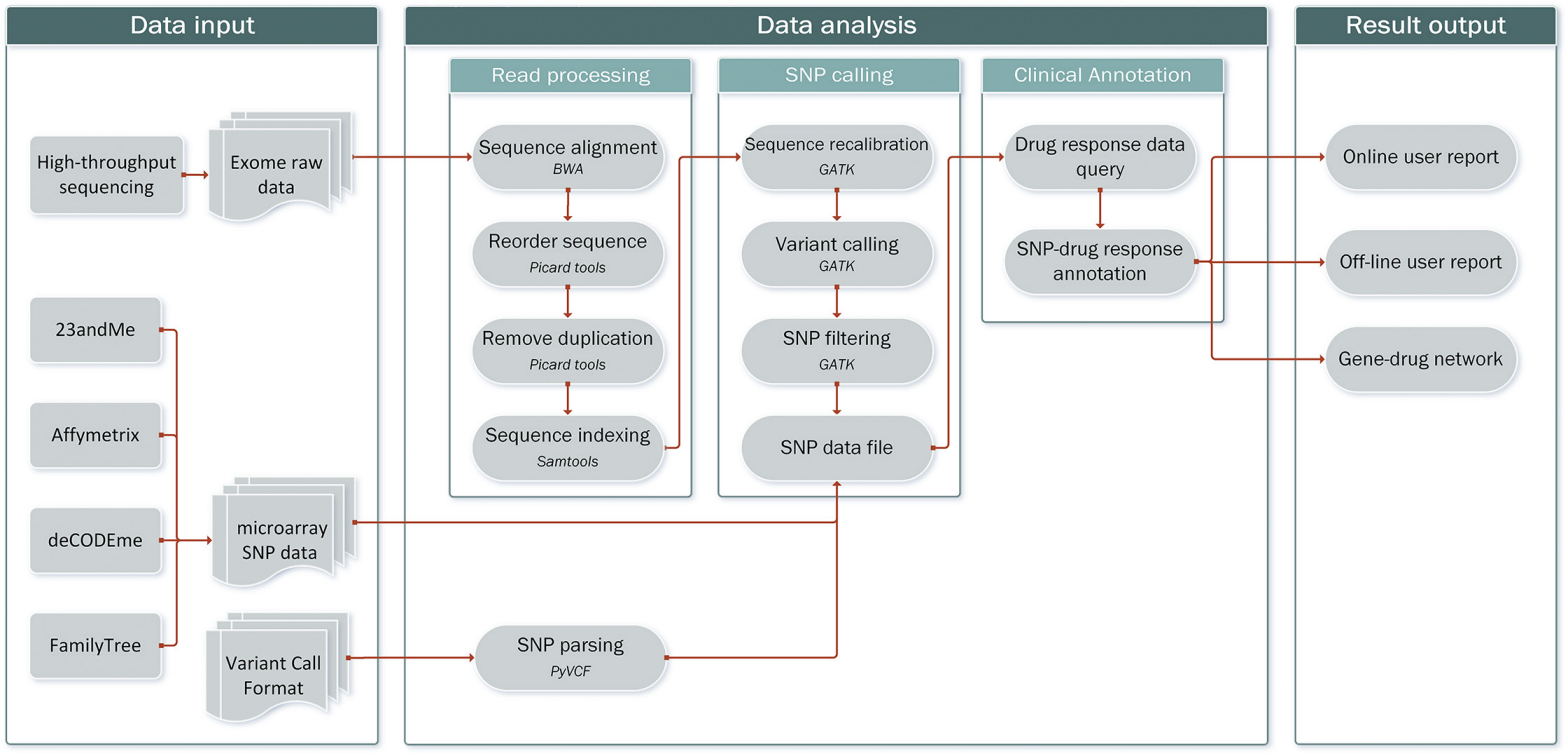
Schematic of the Virtual Pharmacist (VP) workflow. The three components of VP are shown.

The VP workflow has three main components: (i) data input; (ii) annotation and analysis; and (iii) result presentation, consisting of the generation of individual and group annotations and analysis (**Fig 1**). The steps included in the individual curration and analysis modules are described in detail in the accompanying VP developer guide.

### Data input

**Fig 2:**
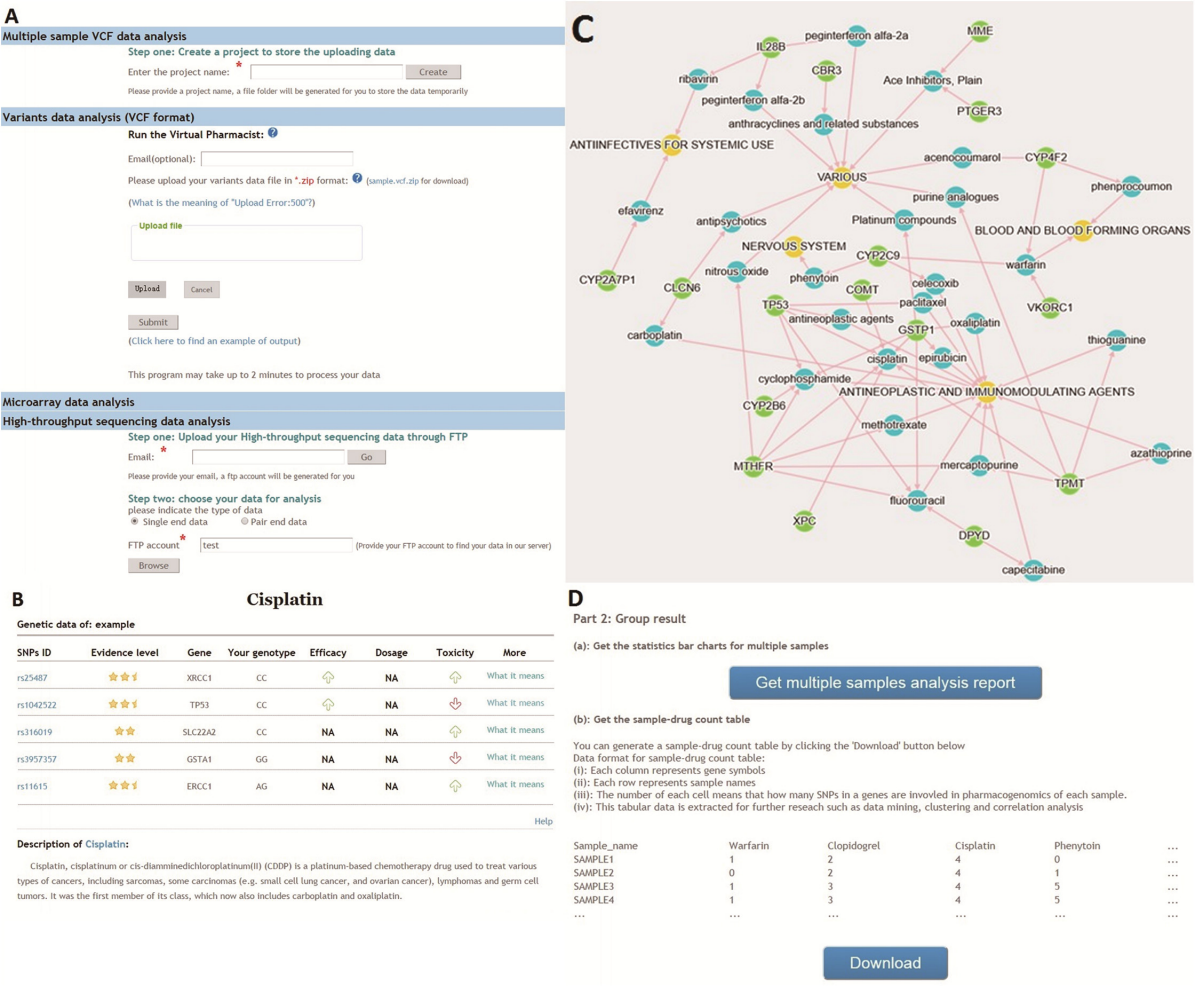
User interface and representative result output of Virtual Pharmacist (VP). **(A**) VP accepts various data format as input; for example, VCF format files, high-throughput sequencing data, and microarray SNP data. (**B**) Cisplatin output as a representative result output. VP reports the SNP ID, evidence level, gene, genotype, efficacy, dosage, toxicity, and detailed description. (**C**) Network view of drug-gene interaction. Green, blue, and yellow circles in the graph represent a specific gene, drug name, and drug category, respectively, based on the DrugBank database. A drug functional classification and a gene are connected if a variant of the gene affects the response of the drug. (**D**) User interface for a representative multiple sample result. VP can analyze multiple VCF data files (each data file should contain just one sample) and calculate the statistics of drug response in a population. Furthermore, VP can provide sample-drug and sample-gene count tables to users for data mining and association studies.

VP accepts Variant Call Format (VCF) files, high-throughput sequencing data, and microarray SNP genotyping data (**Fig 2A**). The VCF specification was developed to store large-scale data from projects such as the 1000 Genomes Project (http://www.1000genomes.org/data). At present VCF-v4.2 is supported in VP. Genotyping data are generated by parsing VCF data with the Python package PyVCF (https://pyvcf.readthedocs.org/en/latest/). SNP id, and chromosome position are also extracted and stored in a simple text format for annotation. Whole-genome/exome sequencing data are usually a few Gigabytes in size, which is too large for webpage uploading. Therefore, we built an FTP server for uploading large genome data files. Users are asked to provide an email address to receive a randomly generated FTP user name and password. Four microarray genotyping data formats (23andMe, Affymetrix, deCODEme, and Family tree DNA) are supported. All data must be uploaded in a compressed zipped archive.

### Read processing and SNP calling

For high-throughput sequencing data in FASTQ format, read alignment and SNP calling are performed.

The sequences are first aligned to the human reference genome (hg19) by BWA(Burrows-Wheeler Alignment tool), which is an ultrafast and memory-efficient sequence aligner [23]. Then, a file in Sequence Alignment/Map (SAM) format is generated with a header section and an alignment section. The alignment results are reordered by the program ReorderSam.jar in Picard Tools package (http://broadinstitute.github.io/picard/). Sequence duplications are marked and removed by the program MarkDuplicates.jar in Picard Tools package to avoid the PCR bias. Then, the alignment results are indexed by SAMTools[24] for SNP calling. Base recalibration based on quality score is performed to reduce false positives in SNP calling. Variants in the recalibrated alignment are called by UnifiedGenotyper in GATK software. The varaints called are further filtered by VariantFiltration program in GATK[25]. All variants are output to a VCF file. The detailed description of command lines and parameters is available in **S2 File.**

### Clinical annotation

Each SNP is mapped to our database containing genotype and phenotype information. Successfully mapped SNPs will be stored in a separate table and output to a report. In the final report, we reported the toxicity, dosages and efficacy of drug response. The influence of SNP on drug response is qualitatively descripted as “increase” or “decrease”. The quantitative information is usually unavailable for most drugs, because the toxicity, dosage and efficacy are affected by multiple factors, such as genotype, disease condition, environment, age, weight, etc.

Because the interactions between drug and target molecular genes are complicated, the efficacy, dosage and toxicity of drugs are studied in different groups using different methodologies. We adopted the evidence level classification method proposed in PharmGKB and assigned every SNP and drug response information to certain evidence level. The detailed description of evidence level can be seen in PharmGKB (https://www.pharmgkb.org/page/clinAnnLevels).

### Database construction

**Fig 3:**
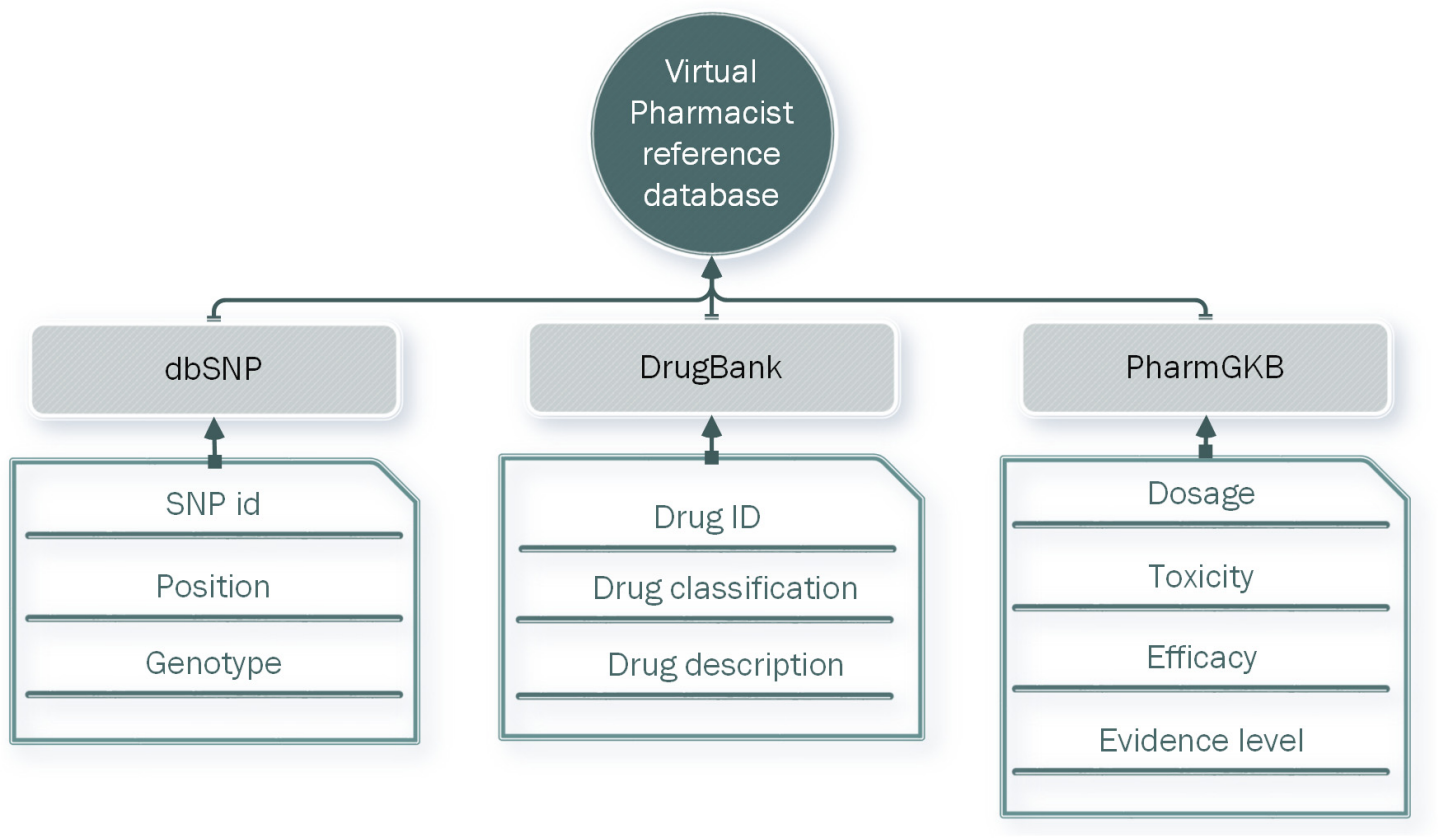
Structure of the reference database of Virtual Pharmacist (VP). Different data sources were integrated to construct the gene-drug interaction reference database.

We constructed a database by integrating annotation information from PharmGKB, DrugBank, and dbSNP (**Fig 3**). A total of 1135 drug-related SNP records corresponding to 193 drugs were collected from PharmGKB [6]. The potential impacts of genetic variations on drug responses such as dosage, toxicity, and efficacy were collected. Genotype and chromosome position information was obtained from dbSNP [1]. Detailed descriptions of the 193 drugs were extracted from DrugBank [22] (S1 Table). The integrated data were stored as a database in a MySQL database management system.

## Results

### Result presentation

Individual and group analysis results are available in VP. The individual analysis result consists of 1) a report that is readily comprehensible to patients and practioners who have basic knowledge in pharmacology, 2) a table that summarizes variants and potential affected drug responses, and 3) visualization of a gene-drug-target network. The group analysis result comprises group statistics by 1) charting distribution of variants and potentially affected drug responses of a target group, 2) a sample-gene variant count table, and 3) a sample-drug count table.

Individual reports contain detailed information about drug response-related SNPs, gene location, the effects on drug efficacy, dosage, and toxicity together with the evidence level of the records at one glance (**Fig 2B**). A sample report file is provided as **S1 File**. Briefly, a drug classification list is provided as the index of the user report. Users can find specific drug-related SNPs simply by clicking the drug name in the index. On each page of the user report, the drug information from Drugbank is presented, followed by user-specific drug-response related SNPs. In particular, the FDA pharmacogenomic biomarker-labeled drug list is highlighted to provide documented information about “drug exposure and clinical response variability, risk for adverse events, genotype-specific dosing, mechanisms of drug action, and polymorphic drug target and disposition genes” (http://www.fda.gov/Drugs/ScienceResearch/ResearchAreas/Pharmacogenetics/ucm083378.htm). A table summary is also provided for quick and easy reference.

### Running time

We tested the running time of VP using various data formats and sizes (**Table 1**). VP took less than 1 minute to process VCF data files.

**Table 1:** VP running time with various input files.

Data type	File size	Uploading time^a^	Running time	CPU
VCF	20 Mb	<1 min	<1 min	1
SNP array	15 Mb	<1 min	<1 min	1
Whole-genome sequencing	111 Gb	379 mins	24 hours	20
Exome sequencing	6.8 Gb	30 mins	3 hours	20

^a^The network uploading speed used for comparison was 5 Mbps.

### Network visualization

To visualize potential pleiotropic and convergent phenotypes, we visualized the relations between genetic variations, drugs, and their targets (**Fig 2C**). VKORC1, CYP2C9, and CYP4F2 are linked with blood-thinning warfarin action, and CYP4F2 also affects another anticoagulant phenprocoumon. CYP2C9 not only modulates anticoagulation, it can also predispose other drug actions that target the nervous system and cancer. For patients with co-morbid conditions, a holistic therapeutic plan may have to be devised. The large number of arrows pointing towards cisplatin in the drug-gene interaction network suggests that its action may be affected by multiple genetic variations. Thus, the network visualization provides users a better overview of the complex network of drug response and genes to help understand the interaction of genomics and drugs.

### Group analysis

The average numbers (mean) and standard deviations of drug-related SNPs for FDA-approved drugs and all drug-related SNPs among five populations are listed. AFR, African; AMR, American; SAS, South Asian; EAS, East Asian; EUR, European.

In the group analysis, VP outputs count tables (**Fig 2D**) that can be used in data mining and trait association studies. To demonstrate the functionalities of the VP group analysis, we retrieved all of the 2504 VCF data files from the 2013 release of 1000 Genomes Project [26] () as an example and detected 291 SNPs associated with differential drug responses. The difference in allele frequency of drug-related SNPs was significant among the five populations (**Table 2** and **S2 Table**), which indirectly indicates that genetic alterations impacting on drug response is different among populations.

**Table 2:** Summary of drug-related SNPs among populations.

	FDA-labeled drug-related SNPs		Drug-related SNPs	
Race	Mean	Standard deviation	Mean	Standard deviation
AFR	56.53	4.10	136.60	8.94
AMR	61.00	4.67	148.96	9.86
SAS	59.95	4.73	142.85	8.98
EAS	59.19	4.00	138.65	8.62
EUR	60.60	5.07	151.20	9.96

**Fig 4:**
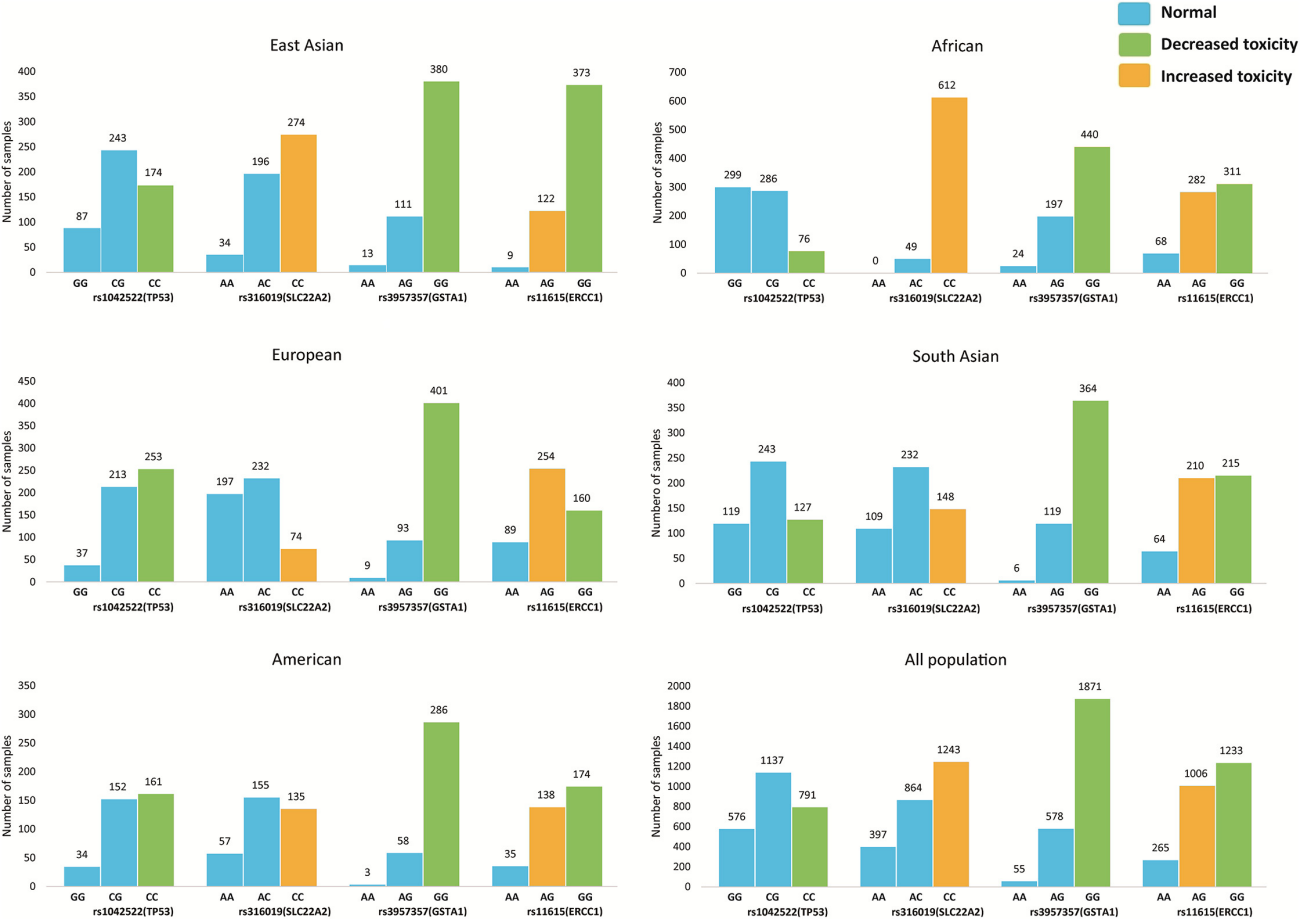
Allele variants of cisplatin-related SNPs and their impacts on cisplatin toxicity. Statistical frequencies of SNPs rs1042522, rs316019, rs11615, and rs3957357 among five major populations from the 1000 Genomes Project are shown together with their impacts on cisplatin toxicity.

We analyzed four SNPs associated with cisplatin toxicity. The distribution of four cisplatin-toxicity associated SNPs for different genotypes among five major human populations is shown in **Fig 4**. Generally, the distribution patterns of SNPs among the populations were significantly different. For instance, the heterozygous genotype of SNP rs1042522 was dominant among the South Asian and East Asian populations, while the homozygous genotype (GG) was dominant in the African population. The homozygous genotype of rs1042522 (CC), which leads to a decreased toxicity for cisplatin, was dominant among the American and European populations. The heterozygous genotype of rs316019 was dominant among European, American, and South Asian populations, and the homozygous genotype (CC///), which might lead to increased toxicity for cisplatin, was dominant among the East Asian and African populations. The heterozygous genotype of rs11615, which leads to increased toxicity of cisplatin, was dominant in the European population, while the homozygous genotype (GG), which might lead to decreased toxicity of cisplatin, was dominant among the other studied populations. In contrast, all the five populations had a similar genotype distribution pattern for rs3957357. We found that the average number of SNPs associated with drug response per individual per population was more than 120; about 60 SNPs were among FDA-labeled pharmacogenomics biomarkers (**Table 2**). These SNPs might indeed modulate therapeutic outcomes.

We also evaluated Watson’s genome [27] and identified 65 drug-related SNPs. Because Watson is Caucasian, we compared his SNPs with the 99 Caucasian SNPs from the 1000 Genomes Project. Moreover, because the existing recommended dosage may have been established before genome sequencing technology was available it is likely that the recommended dosage refers to the most common genotypes. We found that over half (n = 33, **S3 Table**) of Watson’s SNPs were different from the other Caucasian SNPs, including rs2292566, a SNP that is recommended for lower warfarin dosage; and rs1042522 and rs11615, two SNPs associated with increased toxicity of cisplatin. This result highlights the importance of personalized medicine.

## Discussion

We have implemented an integrated pharmacogenomics web service system and made it available in the public repository GitHub. Genome-wide studies have produced large amounts of data and detected a large number of genetic variants. Collecting and integrating annotation information from heterogeneous sources can become the bottleneck that hinders the best use of the abundant information. VP can help fill this gap. Moreover, VP provides group analysis of samples, which extends individual evaluations to possibilities of healthcare policy at higher levels of considerations.

The core data source of VP is PharmGKB, which is a manually curated knowledge base that captures information on genetic variants that can affect drug response. The results from high-throughput studies have created a need to increase the quantity of entries in VP. Therefore, we plan to incorporate information from the PharmacoGenomic Mutation Database [28], which is a more comprehensive collection that was published recently, into the next update in VP.

VP provides individual and group analysis results. In the near future, we will add to VP data mining capabilities such as clustering, pathway or network analysis, as well as integrate information or analysis from related work [29] to allow prediction and more in-depth investigations into the impact of pharmacogenomics at an individual or population level. Our analysis of genomes from the 1000 Genomes Project [26] underlines the heterogeneity of genotype distribution among five different major human populations. Even within the same population, the findings from Watson’s genome [27] highlight the importance of personalized medicine, because membership of a population does not guarantee the same efficacy and safety of a therapy regimen.

VP is packaged with detailed user and developer guides and we can be contacted for assistance related to installation issues usng the email addresses listed on the VP website. Regarding concerns that have been raised over personal data leakage and selling in recent years, our open-source VP is a timely platform that will be conducive to individual evaluations and academic and commercial research.

## Supporting Information

S1 File Sample result report. The comprehensive user report in PDF format is generated automatically by Virtual Pharmacist. It shows the pharmacology of the drug response of genetic variants.
(PDF)

S2 File Supporting information. It includes the detailed description of method for high-throughput data analysis and the strategy for handling SNPs in overlapping genes.
(DOCX)

S1 Table Information for 193 drugs from DrugBank. (XLS)

S2 Table Allele frequency of drug-related SNPs predicted by Virtual Pharmacist among five major populations from the 1000 Genomes Project. (XLS)

S3 Table Summary table of the results for Watson’s genome analyzed by Virtual Pharmacist. A total of 65 variants were predicted to have a genetic impact for drug response, and 33 of them were predicted to have a genetic impact for FDA-approved drug labels.
(XLS)
