# Supplementary material for: Virtual Pharmacist: A Platform for Pharmacogenomics
Source: PLoS One. 2015 Oct 23;10(10):e0141105. doi: 10.1371/journal.pone.0141105 (PMC4619711; doi:10.1371/journal.pone.0141105)
Supplement: S1 File — The comprehensive user report in PDF format is generated automatically by Virtual Pharmacist. It shows the pharmacology of the drug response of genetic variants. (PDF) [file pone.0141105.s001.pdf]

VP Genetic Results

Prepared for: sample1

Jun 1, 2015

Antiinfectives for systemic use 3
- Antivirals for systemic use 3
  - *Efavirenz* 3
  - *Ribavirin* 4

Antineoplastic and immunomodulating agents 4
- Antineoplastic agents 4
  - *Antineoplastic agents* 5
  - *Capecitabine(FDA pharmacogenomic biomarker)* 6
  - *Carboplatin* 7
  - *Celecoxib(FDA pharmacogenomic biomarker)* 8
  - *Cisplatin(FDA pharmacogenomic biomarker)* 9
  - *Cyclophosphamide* 11
  - *Epirubicin* 13
  - *Fluorouracil(FDA pharmacogenomic biomarker)* 14
  - *Mercaptopurine(FDA pharmacogenomic biomarker)* 15
  - *Methotrexate* 16
  - *Oxaliplatin* 18
  - *Paclitaxel* 19
  - *Thioguanine(FDA pharmacogenomic biomarker)* 20
- Immunosuppressive agents 20
  - *Azathioprine(FDA pharmacogenomic biomarker)* 21

Blood and blood forming organs 21
- Antithrombotic agents 21
  - *Phenprocoumon* 22
  - *Warfarin(FDA pharmacogenomic biomarker)* 23

Nervous system 23
- Antiepileptics 23
  - *Phenytoin(FDA pharmacogenomic biomarker)* 24

Various 24
- Unclassified 24
  - *Ace inhibitors, plain* 25
  - *Acenocoumarol* 26
  - *Anthracyclines and related substances* 27
  - *Antipsychotics* 28
  - *Nitrous oxide* 29
  - *Peginterferon alfa-2a* 30
  - *Peginterferon alfa-2b(FDA pharmacogenomic biomarker)* 31
  - *Platinum compounds* 32
  - *Purine analogues* 33

Efavirenz

For HIV infection that has not previously been treated, efavirenz and lamivudine in combination with zidovudine or tenofovir is the preferred NNRTI-based regimen.

Your genetic data

SNPs ID	Evidence level	Gene	Your genotype	Efficacy	Dosage	Toxicity
rs3745274	★★★	CYP2A7P1	GT	NA	NA	increased

For a list of references for each variant, please see the freely available VP on the Internet https://www.sustc-genome.org.cn/vp

The description of **rs3745274**

Patients with the GT genotype may have an increased risk of efavirenz-induced side effects (central nervous symptoms, fatigue, sleep disorder, liver toxicity or early termination of drug therapy) as compared to patients with the GG genotype. Other genetic and clinical factors may also influence a patients risk for toxicity.

Ribavirin

A nucleoside antimetabolite antiviral agent that blocks nucleic acid synthesis and is used against both RNA and DNA viruses. [PubChem]

Your genetic data

SNPs ID	Evidence level	Gene	Your genotype	Efficacy	Dosage	Toxicity
rs12979860	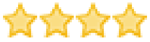	IL28B	TT	decreased	NA	NA
rs8099917	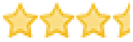	IL28B	GG	decreased	NA	NA

For a list of references for each variant, please see the freely available VP on the Internet https://www.sustc-genome.org.cn/vp

The description of **rs12979860**

Patients with the TT genotype may have decreased response to peginterferon alpha and ribavirin in people with Hepatitis C genotype 1 as compared to patients with the CC genotype. Patients with the TT genotype may also have lower spontaneous clearance in acute HCV infections than patients with the CC genotype. Other genetic and clinical factors may also influence a patients response to peginterferon.

The description of **rs8099917**

Patients with the GG genotype may have decreased response (lower SVR) to peginterferon alfa and ribavirin therapy in people with Chronic Hepatitis C as compared to patients with the TT genotype. Other genetic and clinical factors may also influence a patients response to peginterferon alfa and ribavirin therapy.

Antineoplastic agents

Inhibiting or preventing development of neoplasms; checking maturation and proliferation of malignant cells

Your genetic data

SNPs ID	Evidence level	Gene	Your genotype	Efficacy	Dosage	Toxicity
rs1042522	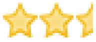	TP53	CG	NA	NA	increased

For a list of references for each variant, please see the freely available VP on the Internet https://www.sustc-genome.org.cn/vp

The description of **rs1042522**

Patients with the CG genotype may have 1) increased risk for toxicity 2) decreased survival when treated with antineoplastic agents as compared to patients with the CC genotype. Other genetic and clinical factors may also influence a patients response to antineoplastic agents.

Capecitabine(FDA pharmacogenomic biomarker)

Capecitabine is an orally-administered chemotherapeutic agent used in the treatment of metastatic breast and colorectal cancers. Capecitabine is a prodrug, that is enzymatically converted to fluorouracil (antimetabolite) in the tumor, where it inhibits DNA synthesis and slows growth of tumor tissue.

Your genetic data

SNPs ID	Evidence level	Gene	Your genotype	Efficacy	Dosage	Toxicity
rs2297595	★★★	DPYD	CC	NA	NA	increased

For a list of references for each variant, please see the freely available VP on the Internet https://www.sustc-genome.org.cn/vp

The description of **rs2297595**

Patients with the CC genotype may have increased risk of severe toxicity when treated with fluoropyrimidines as compared to patients with the TT genotype. Other genetic and clinical factors may also influence a patients chance of adverse events.

Carboplatin

An organoplatinum compound that possesses antineoplastic activity.

Your genetic data

SNPs ID	Evidence level	Gene	Your genotype	Efficacy	Dosage	Toxicity
rs1801133	★★★	CLCN6	AA	increased	NA	increased

For a list of references for each variant, please see the freely available VP on the Internet https://www.sustc-genome.org.cn/vp

The description of **rs1801133**

Patients with AA genotype may have 1) increased likelihood of response 2) increased progression free survival to carboplatin in people with Non-Small-Cell Lung Carcinoma as compared to patients with genotype AG or GG. Other genetic and clinical factors may also influence a patients response to carboplatin.

Celecoxib(FDA pharmacogenomic biomarker)

Celecoxib is a non-steroidal anti-inflammatory drug

Your genetic data

SNPs ID	Evidence level	Gene	Your genotype	Efficacy	Dosage	Toxicity
rs1799853	⭐⭐	CYP2C9	CT	decreased	NA	NA

For a list of references for each variant, please see the freely available VP on the Internet https://www.sustc-genome.org.cn/vp

The description of **rs1799853**

Patients with the CT genotype who are treated with celecoxib may have a decreased metabolism of celecoxib and as compared to patients with the CC genotype. This association has been contradicted in some studies. Other genetic and clinical factors may also influence a patients response to celecoxib.

Cisplatin(FDA pharmacogenomic biomarker)

Cisplatin, cisplatinum or cis-diamminedichloroplatinum(II) (CDDP) is a platinum-based chemotherapy drug used to treat various types of cancers, including sarcomas, some carcinomas (e.g. small cell lung cancer, and ovarian cancer), lymphomas and germ cell tumors. It was the first member of its class, which now also includes carboplatin and oxaliplatin.

Your genetic data

SNPs ID	Evidence level	Gene	Your genotype	Efficacy	Dosage	Toxicity
rs2228001	★★★½	XPC	TT	NA	NA	decreased
rs1800460	★★	TPMT	CT	NA	NA	increased
rs1042522	★★½	TP53	CG	NA	NA	increased
rs1801133	★★	MTHFR	AA	increased	NA	increased
rs1695	★★	GSTP1	AG	decreased	NA	NA
rs1695	★★	GSTP1	AG	decreased	NA	NA
rs9332377	★★★½	COMT	CT	NA	NA	increased

For a list of references for each variant, please see the freely available VP on the Internet https://www.sustc-genome.org.cn/vp

The description of **rs2228001**
Patients with the TT genotype may have a decreased but not non-existent risk for toxicity with cisplatin treatment as compared to patients with the GG or GT genotype. Other genetic and clinical factors may also influence a patients risk for toxicity.

The description of **rs1800460**
Patients with the TC genotype and cancer who are treated with cisplatin may have an increased risk for hearing loss as compared to patients with the CC genotype. Other genetic and clinical factors may also influence a patients risk for hearing loss with cisplatin treatment.

The description of **rs1042522**
Patients with the CG genotype may have 1) increased risk for toxicity 2) decreased survival when treated with antineoplastic agents as compared to patients with the CC genotype. Other genetic and clinical factors may also influence a patients response to antineoplastic agents.

The description of **rs1801133**
Patients with the AA genotype may have: 1) increased likelihood of response to chemotherapy, 2) increased likelihood of Drug Toxicity when treated with cisplatin in cancer patients as compared to patients with genotypes AG or GG. Other genetic and clinical factors may also influence a patients response to cisplatin.

The description of **rs1695**
Patients with the AG genotype and Ovarian Neoplasms who are treated with cisplatin and cyclophosphamide may have a decreased likelihood of progression free survival as compared to patients with the AA genotype. However,

this association was contradicted in other studies. Other genetic and clinical factors may also influence a patients response to cisplatin and cyclophosphamide treatment.

The description of **rs1695**
Patients with the AG genotype and Ovarian Neoplasms who are treated with cisplatin and cyclophosphamide may have a decreased progression free survival as compared to patients with the AA genotype. This association is contradicted in other studies. Other genetic and clinical factors may also influence a patients response to cisplatin and cyclophosphamide.

The description of **rs9332377**
Patients with the CT genotype who are treated with cisplatin may have an increased risk of hearing loss as compared to patients with the CC genotype. Other genetic and clinical factors may also influence a patients risk for hearing loss with cisplatin treatment.

Cyclophosphamide

Precursor of an alkylating nitrogen mustard antineoplastic and immunosuppressive agent that must be activated in the liver to form the active aldophosphamide. It has been used in the treatment of lymphoma and leukemia. Its side effect, alopecia, has been used for defleecing sheep. Cyclophosphamide may also cause sterility, birth defects, mutations, and cancer. [PubChem]

Your genetic data

SNPs ID	Evidence level	Gene	Your genotype	Efficacy	Dosage	Toxicity
rs1042522		TP53	CG	NA	NA	increased
rs1801133		MTHFR	AA	NA	NA	increased
rs1695		GSTP1	AG	increased	NA	NA
rs1695		GSTP1	AG	decreased	NA	NA
rs1695		GSTP1	AG	decreased	NA	NA
rs3745274		CYP2B6	GT	NA	NA	decreased
rs3745274		CYP2B6	GT	NA	decreased	NA

For a list of references for each variant, please see the freely available VP on the Internet https://www.sustc-genome.org.cn/vp

The description of **rs1042522**

Patients with the CG genotype may have 1) increased risk for toxicity 2) decreased survival when treated with antineoplastic agents as compared to patients with the CC genotype. Other genetic and clinical factors may also influence a patients response to antineoplastic agents.

The description of **rs1801133**

Patients with the AA genotype may have increased likelihood of Drug Toxicity when treated with cyclophosphamide as compared to patients with the GG or AG genotype. Other genetic and clinical factors may also influence a patients risk of toxicity to cyclophosphamide.

The description of **rs1695**

Patients with the AG genotype and Breast Neoplasms who are treated with cyclophosphamide and epirubicin may have 1) increased drug response 2) decreased severity of toxicity as compared to patients with GG genotype. Some patients were additionally treated with fluorouracilOther genetic and clinical factors may influence a patients response to cyclophosphamide, epirubicin and fluorouracil.

The description of **rs1695**

Patients with the AG genotype and Ovarian Neoplasms who are treated with cisplatin and cyclophosphamide may have a decreased likelihood of progression free survival as compared to patients with the AA genotype. However, this association was contradicted in other studies. Other genetic and clinical factors may also influence a patients response to cisplatin and cyclophosphamide treatment.

The description of **rs1695**

Patients with the AG genotype and Ovarian Neoplasms who are treated with cisplatin and cyclophosphamide may have a decreased progression free survival as compared to patients with the AA genotype. This association is contradicted in other studies. Other genetic and clinical factors may also influence a patients response to cisplatin and cyclophosphamide.

The description of **rs3745274**
Leukemia patients who are recipients of HLA-identical hematopoietic stem cell transplantation from donors with the GT genotype may have a decreased risk of developing veno-occlusive disease of the liver when treated with cyclophosphamide as compared to donor cells with the GG genotype. Other genetic and clinical factors may also influence a patients risk for venoocclusive disease of the liver.

The description of **rs3745274**
Patients with the GT genotype and Breast Cancer who are treated with cyclophosphamide and doxorubicin may be more likely to require a reduction in dose as compared to patients with the TT genotype, or may be less likely to require a reduction in dose as compared to patients with the GG genotype. Other genetic and clinical factors may also influence a patients dose requirements.

Epirubicin

The compound exerts its antitumor effects by interference with the synthesis and function of DNA.

Your genetic data

SNPs ID	Evidence level	Gene	Your genotype	Efficacy	Dosage	Toxicity
rs1695	⭐⭐⭐	GSTP1	AG	increased	NA	NA

For a list of references for each variant, please see the freely available VP on the Internet https://www.sustc-genome.org.cn/vp

The description of **rs1695**

Patients with the AG genotype and Breast Neoplasms who are treated with cyclophosphamide and epirubicin may have 1) increased drug response 2) decreased severity of toxicity as compared to patients with GG genotype. Some patients were additionally treated with fluorouracilOther genetic and clinical factors may influence a patients response to cyclophosphamide, epirubicin and fluorouracil.

Fluorouracil(FDA pharmacogenomic biomarker)

A pyrimidine analog that is an antineoplastic antimetabolite. It interferes with DNA synthesis by blocking the thymidylate synthetase conversion of deoxyuridylic acid to thymidylic acid. [PubChem]

Your genetic data

SNPs ID	Evidence level	Gene	Your genotype	Efficacy	Dosage	Toxicity
rs1042522	★★½	TP53	CG	NA	NA	increased
rs1801133	★★★	MTHFR	AA	NA	NA	increased
rs1695	★★	GSTP1	AG	NA	NA	decreased
rs2297595	★★★	DPYD	CC	NA	NA	increased

For a list of references for each variant, please see the freely available VP on the Internet https://www.sustc-genome.org.cn/vp

The description of **rs1042522**

Patients with the CG genotype may have 1) increased risk for toxicity 2) decreased survival when treated with antineoplastic agents as compared to patients with the CC genotype. Other genetic and clinical factors may also influence a patients response to antineoplastic agents.

The description of **rs1801133**

Patients with the AA genotype may have increased risk of Drug Toxicity in cancer patients treated with fluorouracil-based therapy as compared to patients with the GG genotype. However, conflicting finding of no-association for this genotype has also been reported. Other genetic and clinical factors may also influence a patients risk of toxicity to fluorouracil.

The description of **rs1695**

Patients with the AG genotype and cancer who are treated with fluorouracil may have a lower, but not absent, risk of hematological toxicity as compared to patients with the AA genotype, or may have a higher risk of hematological toxicity as compared to patients with the GG genotype. Other genetic and clinical factors may also influence a patients risk for hematological toxicity when exposed to fluorouracil.

The description of **rs2297595**

Patients with the CC genotype may have increased risk of severe toxicity when treated with fluoropyrimidines as compared to patients with the TT genotype. Other genetic and clinical factors may also influence a patients chance of adverse events.

Mercaptopurine(FDA pharmacogenomic biomarker)

An antimetabolite antineoplastic agent with immunosuppressant properties. It interferes with nucleic acid synthesis by inhibiting purine metabolism and is used, usually in combination with other drugs, in the treatment of or in remission maintenance programs for leukemia. [PubChem]

Your genetic data

SNPs ID	Evidence level	Gene	Your genotype	Efficacy	Dosage	Toxicity
rs1800460	★★★★	TPMT	CT	NA	NA	increased
rs1800462	★★★★	TPMT	GG	NA	NA	increased
rs1801133	★★	MTHFR	AA	NA	NA	NA

For a list of references for each variant, please see the freely available VP on the Internet https://www.sustc-genome.org.cn/vp

The description of **rs1800460**
Patients with the CT genotype may have an increased risk for toxicity with thiopurine drugs and purine analogues as compared to patients with the CC genotype. Other genetic and clinical factors may also influence a patients risk for toxicity.

The description of **rs1800462**
Patients with the GG genotype (TPMT*2/*2): 1) may decreased deactivation of thiopurines 2) may have an increased risk for toxicity to thiopurines as compared to patients with the CC genotype (e.g. TPMT*1/*1). Other genetic and clinical factors may also influence a patients risk for toxicity.

The description of **rs1801133**
Patients with the AA genotype may have increased likelihood of treatment interruptions when treated with mercaptopurine in people with Precursor Cell Lymphoblastic Leukemia-Lymphoma as compared to patients with genotype GG. However, contradictory finding has been reported. Other genetic and clinical factors may also influence a patients risk for toxicity to mercaptopurine.

Methotrexate

An antineoplastic antimetabolite with immunosuppressant properties.

Your genetic data

SNPs ID	Evidence level	Gene	Your genotype	Efficacy	Dosage	Toxicity
rs1801133	★★★½	MTHFR	AA	NA	NA	increased
rs1801133	★★	MTHFR	AA	NA	NA	increased
rs1801133	★★	MTHFR	AA	NA	NA	increased
rs1801133	★★	MTHFR	AA	decreased	NA	NA
rs1801133	★★	MTHFR	AA	decreased	decreased	increased
rs1801133	★★	MTHFR	AA	NA	NA	decreased

For a list of references for each variant, please see the freely available VP on the Internet https://www.sustc-genome.org.cn/vp

The description of **rs1801133**
Patients with the AA genotype with Leukemia or Lymphoma who are treated with methotrexate regimens may have an increased risk and increased severity of mucositis, as compared to patients with the GA or GG genotype. Other genetic and clinical factors may also influence a patients risk of oral mucositis.

The description of **rs1801133**
Patients with the AA genotype and Arthritis who are treated with methotrexate may have an increased risk of adverse events and toxicity as compared to patients with the GG genotype (though this association has not been found in all studies). There does not seem to be an association between this genotype and response to methotrexate treatment. Other genetic and clinical factors may also influence a patients risk for adverse events with methotrexate treatment.

The description of **rs1801133**
Patients with the AA genotype and non-Hodgkin lymphoma who are treated with methotrexate may have an increased risk of mucositis, thrombocytopenia and hepatic toxicity as compared to patients with the GG genotype. This association was not found in pediatric patients with non-Hodgkin lymphoma who were treated with methotrexate. Other genetic and clinical factors may also influence a patients risk for methotrexate-induced toxicity.

The description of **rs1801133**
Patients with the AA genotype with non-hodgkin lymphoma who are treated with methotrexate may be less likely to have event free survival at 5 years as compared to patients with the GG genotype. This genotype was not associated with treatment outcome in pediatric patients with non-hodgkin lymphoma as compared to the GG genotype. Other genetic and clinical factors may also influence a patients response to methotrexate treatment.

The description of **rs1801133**
Patients with the AA genotype and Leukemia who are treated with methotrexate: 1) may have poorer response to treatment 2) may be at increased risk of toxicity 3) may require a lower dose of methotrexate as compared to patients with the GG genotype. This association has been contradicted or not found in several studies. Other

genetic and clinical factors may also influence a patients risk for toxicity and response with methotrexate treatment.

The description of **rs1801133**
Patients with the AA genotype and leukemia who undergo hematopoietic cell transplant and are treated with methotrexate may have a decreased risk of Graft vs Host disease as compared to patients with the GG genotype. Other genetic and clinical factors may also influence a patients risk Graft vs Host disease and efficacy of methotrexate treatment.

Oxaliplatin

Oxaliplatin is a platinum-based chemotherapy drug in the same family as cisplatin and carboplatin. It is typically administered in combination with fluorouracil and leucovorin in a combination known as Folfox for the treatment of colorectal cancer. Compared to cisplatin the two amine groups are replaced by cyclohexyldiamine for improved antitumour activity.

Your genetic data

SNPs ID	Evidence level	Gene	Your genotype	Efficacy	Dosage	Toxicity
rs1695	⭐⭐	GSTP1	AG	NA	NA	increased

For a list of references for each variant, please see the freely available VP on the Internet https://www.sustc-genome.org.cn/vp

The description of **rs1695**

Patients with the AG genotype and cancer who are treated with oxaliplatin or platinum compounds may have a decreased, but not absent, risk for hematological toxicity, neurotoxicity, neutropenia, and discontinuation of treatment as compared to patients with the AA genotype. Conflicting data exist for the neurotoxicity risk showing that patients with the AG might have an increased risk. Other genetic and clinical factors may also influence a patients risk for adverse events with oxaliplatin or platinum compounds treatment.

Paclitaxel

A cyclodecane isolated from the bark of the Pacific yew tree, TAXUS brevifolia. It stabilizes microtubules in their polymerized form leading to cell death. [PubChem] ABI-007 (Abraxane) is the latest attempt to improve upon paclitaxel, one of the leading chemotherapy treatments.

Your genetic data

SNPs ID	Evidence level	Gene	Your genotype	Efficacy	Dosage	Toxicity
rs1042522		TP53	CG	NA	NA	increased

For a list of references for each variant, please see the freely available VP on the Internet https://www.sustc-genome.org.cn/vp

The description of **rs1042522**

Patients with the CG genotype may have 1) increased risk for toxicity 2) decreased survival when treated with antineoplastic agents as compared to patients with the CC genotype. Other genetic and clinical factors may also influence a patients response to antineoplastic agents.

Thioguanine(FDA pharmacogenomic biomarker)

An antineoplastic compound which also has antimetabolite action. The drug is used in the therapy of acute leukemia.

Your genetic data

SNPs ID	Evidence level	Gene	Your genotype	Efficacy	Dosage	Toxicity
rs1800460	★★★★	TPMT	CT	NA	NA	increased
rs1800462	★★★★	TPMT	GG	NA	NA	increased

For a list of references for each variant, please see the freely available VP on the Internet https://www.sustc-genome.org.cn/vp

The description of **rs1800460**

Patients with the CT genotype may have an increased risk for toxicity with thiopurine drugs and purine analogues as compared to patients with the CC genotype. Other genetic and clinical factors may also influence a patients risk for toxicity.

The description of **rs1800462**

Patients with the GG genotype (TPMT*2/*2): 1) may decreased deactivation of thiopurines 2) may have an increased risk for toxicity to thiopurines as compared to patients with the CC genotype (e.g. TPMT*1/*1). Other genetic and clinical factors may also influence a patients risk for toxicity.

Azathioprine(FDA pharmacogenomic biomarker)

An immunosuppressive pro-drug. It is converted into 6-mercaptopurine in the body where it blocks purine metabolism and DNA synthesis.

Your genetic data

SNPs ID	Evidence level	Gene	Your genotype	Efficacy	Dosage	Toxicity
rs1800460	★★★★	TPMT	CT	NA	NA	increased
rs1800462	★★★★	TPMT	GG	NA	NA	increased

For a list of references for each variant, please see the freely available VP on the Internet https://www.sustc-genome.org.cn/vp

The description of **rs1800460**

Patients with the CT genotype may have an increased risk for toxicity with thiopurine drugs and purine analogues as compared to patients with the CC genotype. Other genetic and clinical factors may also influence a patients risk for toxicity.

The description of **rs1800462**

Patients with the GG genotype (TPMT*2/*2): 1) may decreased deactivation of thiopurines 2) may have an increased risk for toxicity to thiopurines as compared to patients with the CC genotype (e.g. TPMT*1/*1). Other genetic and clinical factors may also influence a patients risk for toxicity.

Phenprocoumon

Coumarin derivative that acts as a long acting oral anticoagulant. [PubChem]

Your genetic data

SNPs ID	Evidence level	Gene	Your genotype	Efficacy	Dosage	Toxicity
rs2108622		CYP4F2	TT	NA	increased	NA

For a list of references for each variant, please see the freely available VP on the Internet https://www.sustc-genome.org.cn/vp

The description of **rs2108622**

Patients with the TT genotype who are taking an oral anticoagulant may require the highest dose as compared to patients with the CT or TT genotype. Other genetic and clinical factors may also influence a patients required dose.

Warfarin(FDA pharmacogenomic biomarker)

Warfarin is an anticoagulant drug normally used to prevent blood clot formation as well as migration. Although originally marketed as a pesticide (d-Con, Rodex, among others), Warfarin has since become the most frequently prescribed oral coagulant in North America. Warfarin has several properties that should be noted when used medicinally, including its ability to cross the placental barrier during pregnancy which can result in fetal bleeding, spontaneous abortion, preterm birth, stillbirth, and neonatal death. Additional adverse effects such as necrosis, purple toe syndrome, osteoporosis, valve and artery calcification, and drug interactions have also been documented with warfarin use. Warfarin does not actually affect blood viscosity, rather, it inhibits vitamin-k dependent synthesis of biologically active forms of various clotting factors in addition to several regulatory factors.

Your genetic data

SNPs ID	Evidence level	Gene	Your genotype	Efficacy	Dosage	Toxicity
rs7294	★★★½	VKORC1	CT	NA	increased	NA
rs2108622	★★★½	CYP4F2	TT	NA	increased	NA
rs1799853	★★★★	CYP2C9	CT	NA	decreased	NA

For a list of references for each variant, please see the freely available VP on the Internet https://www.sustc-genome.org.cn/vp

The description of **rs7294**
Patients with the CT genotype who are treated with warfarin may require a higher dose as compared to patients with the CC genotype.

The description of **rs2108622**
Patients with the TT genotype who are taking an oral anticoagulant may require the highest dose as compared to patients with the CT or TT genotype. Other genetic and clinical factors may also influence a patients required dose.

The description of **rs1799853**
Patients with the CT genotype who are treated with warfarin may require a lower dose as compared to patients with the CC genotype. Other genetic and clinical factors may also influence a patients dose of warfarin.

Phenytoin(FDA pharmacogenomic biomarker)

An anticonvulsant that is used in a wide variety of seizures. It is also an anti-arrhythmic and a muscle relaxant. The mechanism of therapeutic action is not clear, although several cellular actions have been described including effects on ion channels, active transport, and general membrane stabilization.

Your genetic data

SNPs ID	Evidence level	Gene	Your genotype	Efficacy	Dosage	Toxicity
rs1799853	⭐⭐	CYP2C9	CT	NA	NA	increased

For a list of references for each variant, please see the freely available VP on the Internet https://www.sustc-genome.org.cn/vp

The description of **rs1799853**

Patients with the CT genotype and Epilepsy who are treated with phenytoin may have a decreased metabolism of phenytoin, increased plasma free phenytoin concentration, and an increased risk of drug toxicity as compared to patients with the CC genotype. These associations have been contradicted in some studies. Other genetic and clinical factors may also influence a patients response to phenytoin.

Ace inhibitors, plain

An ACE inhibitor (or angiotensin-converting-enzyme inhibitor) is a pharmaceutical drug used primarily for the treatment of hypertension (elevated blood pressure) and congestive heart failure.

Your genetic data

SNPs ID	Evidence level	Gene	Your genotype	Efficacy	Dosage	Toxicity
rs11209716	★★	PTGER3	CC	NA	NA	decreased
rs2016848	★★	MME	AA	NA	NA	increased

For a list of references for each variant, please see the freely available VP on the Internet https://www.sustc-genome.org.cn/vp

The description of **rs11209716**

Patients with the CC genotype and hypertension who are treated with ACE inhibitors may have a decreased, but not absent, risk of cough as compared to patients with the TT genotype. Other genetic and clinical factors may also influence a patients risk of cough when treated with ACE inhibitors.

The description of **rs2016848**

Patients with the AA genotype who are treated with ACE inhibitors may have an increased risk for cough as compared to patients with the GG genotype. Other genetic and clinical factors may also influence a patients risk for cough with ACE inhibitor treatment.

Acenocoumarol

Acenocoumarol is an anticoagulant that functions as a vitamin K antagonist (like warfarin). It is a derivative of coumarin and is marketed under the brand names Sintrom and Sinthrome.

Your genetic data

SNPs ID	Evidence level	Gene	Your genotype	Efficacy	Dosage	Toxicity
rs2108622	★★★½	CYP4F2	TT	NA	increased	NA

For a list of references for each variant, please see the freely available VP on the Internet https://www.sustc-genome.org.cn/vp

The description of **rs2108622**

Patients with the TT genotype who are taking an oral anticoagulant may require the highest dose as compared to patients with the CT or TT genotype. Other genetic and clinical factors may also influence a patients required dose.

Anthracyclines and related substances

These compounds are used to treat many cancers, including leukemias, lymphomas, breast, uterine, ovarian, and lung cancers.

Your genetic data

SNPs ID	Evidence level	Gene	Your genotype	Efficacy	Dosage	Toxicity
rs1056892	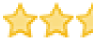	CBR3	AA	NA	NA	decreased

For a list of references for each variant, please see the freely available VP on the Internet https://www.sustc-genome.org.cn/vp

The description of **rs1056892**

Patients with the AA genotype may have decreased risk of cardiac damage after anthracycline exposure as compared to patients with the GG genotype. Patients with the AA genotype may still be at risk for adverse events when exposed to anthracyclines based on their genotype. Other genetic and clinical factors may also influence a patients risk for adverse events.

Antipsychotics

Antipsychotics (also known as neuroleptics or major tranquilizers)[1] are a class of psychiatric medication primarily used to manage psychosis (including delusions, hallucinations, or disordered thought), in particular in schizophrenia and bipolar disorder, and is increasingly being used in the management of non-psychotic disorders (ATC code N05A).

Your genetic data

SNPs ID	Evidence level	Gene	Your genotype	Efficacy	Dosage	Toxicity
rs1801133	⭐⭐	CLCN6	AA	NA	NA	increased

For a list of references for each variant, please see the freely available VP on the Internet https://www.sustc-genome.org.cn/vp

The description of **rs1801133**

Patients with the AA genotype treated with antipsychotics may have increased risk for metabolic syndrome as compared to patients with the GG genotype. Other genetic and clinical factors may also influence a patients risk for adverse events.

Nitrous oxide

Nitrous oxide, commonly known as laughing gas, nitrous, nitro, or NOS[1] is a chemical compound

Your genetic data

SNPs ID	Evidence level	Gene	Your genotype	Efficacy	Dosage	Toxicity
rs1801133	★★	MTHFR	AA	NA	NA	NA

For a list of references for each variant, please see the freely available VP on the Internet https://www.sustc-genome.org.cn/vp

The description of **rs1801133**

Patients with the AA genotype who undergo elective surgery with nitrous oxide anesthesia may have higher plasma total homocysteine concentrations as compared to patients with the GG genotype. Other genetic and clinical factors may also influence a patients homocysteine levels after nitrous oxide anesthesia.

Peginterferon alfa-2a

Pegylated interferon alfa-2a (pegylated with a branched 40 kDa PEG chain; commercial name Pegasys) is an antiviral drug discovered at the pharmaceutical company F. Hoffmann-La Roche; it has a dual mode of action - both antiviral and on the immune system. The addition of polyethylene glycol to the interferon, through a process known as pegylation, enhances the half-life of the interferon when compared to its native form.

Your genetic data

SNPs ID	Evidence level	Gene	Your genotype	Efficacy	Dosage	Toxicity
rs12979860	★★★★	IL28B	TT	decreased	NA	NA
rs8099917	★★★½	IL28B	GG	decreased	NA	NA

For a list of references for each variant, please see the freely available VP on the Internet https://www.sustc-genome.org.cn/vp

The description of **rs12979860**

Patients with the TT genotype may have decreased response to peginterferon alpha and ribavirin in people with Hepatitis C genotype 1 as compared to patients with the CC genotype. Patients with the TT genotype may also have lower spontaneous clearance in acute HCV infections than patients with the CC genotype. Other genetic and clinical factors may also influence a patients response to peginterferon.

The description of **rs8099917**

Patients with the GG genotype may have decreased response (lower SVR) to peginterferon alfa and ribavirin therapy in people with Chronic Hepatitis C as compared to patients with the TT genotype. Other genetic and clinical factors may also influence a patients response to peginterferon alfa and ribavirin therapy.

Peginterferon alfa-2b(FDA pharmacogenomic biomarker)

Pegylated interferon alfa-2a (pegylated with a branched 40 kDa PEG chain; commercial name Pegasys) is an antiviral drug discovered at the pharmaceutical company F. Hoffmann-La Roche; it has a dual mode of action - both antiviral and on the immune system. The addition of polyethylene glycol to the interferon, through a process known as pegylation, enhances the half-life of the interferon when compared to its native form.

Your genetic data

SNPs ID	Evidence level	Gene	Your genotype	Efficacy	Dosage	Toxicity
rs12979860	★★★★	IL28B	TT	decreased	NA	NA
rs8099917	★★★½	IL28B	GG	decreased	NA	NA

For a list of references for each variant, please see the freely available VP on the Internet https://www.sustc-genome.org.cn/vp

The description of **rs12979860**

Patients with the TT genotype may have decreased response to peginterferon alpha and ribavirin in people with Hepatitis C genotype 1 as compared to patients with the CC genotype. Patients with the TT genotype may also have lower spontaneous clearance in acute HCV infections than patients with the CC genotype. Other genetic and clinical factors may also influence a patients response to peginterferon.

The description of **rs8099917**

Patients with the GG genotype may have decreased response (lower SVR) to peginterferon alfa and ribavirin therapy in people with Chronic Hepatitis C as compared to patients with the TT genotype. Other genetic and clinical factors may also influence a patients response to peginterferon alfa and ribavirin therapy.

Platinum compounds

Your genetic data

SNPs ID	Evidence level	Gene	Your genotype	Efficacy	Dosage	Toxicity
rs1695	⭐⭐	GSTP1	AG	NA	NA	increased

For a list of references for each variant, please see the freely available VP on the Internet https://www.sustc-genome.org.cn/vp

The description of **rs1695**

Patients with the AG genotype and cancer who are treated with oxaliplatin or platinum compounds may have a decreased, but not absent, risk for hematological toxicity, neurotoxicity, neutropenia, and discontinuation of treatment as compared to patients with the AA genotype. Conflicting data exist for the neurotoxicity risk showing that patients with the AG might have an increased risk. Other genetic and clinical factors may also influence a patients risk for adverse events with oxaliplatin or platinum compounds treatment.

Purine analogues

Purine analogues are antimetabolites that mimic the structure of metabolic purines. Including Mercaptopurine, Thioguanine, Fludarabine

Your genetic data

SNPs ID	Evidence level	Gene	Your genotype	Efficacy	Dosage	Toxicity
rs1800460	★★★★	TPMT	CT	NA	NA	increased
rs1800462	★★★★	TPMT	GG	NA	NA	increased

For a list of references for each variant, please see the freely available VP on the Internet https://www.sustc-genome.org.cn/vp

The description of **rs1800460**

Patients with the CT genotype may have an increased risk for toxicity with thiopurine drugs and purine analogues as compared to patients with the CC genotype. Other genetic and clinical factors may also influence a patients risk for toxicity.

The description of **rs1800462**

Patients with the GG genotype (TPMT*2/*2): 1) may decreased deactivation of thiopurines 2) may have an increased risk for toxicity to thiopurines as compared to patients with the CC genotype (e.g. TPMT*1/*1). Other genetic and clinical factors may also influence a patients risk for toxicity.

The overview of VP

VP which is developed by He lab in the South University of Science and Technology of China is an online tool that interprets personal genome for the impact of genetic variation on drug response. Base on the carefully selected data from international and authoritative includinig PharmGKB, dbSNP and DrugBank?we can take high-throughput sequencing raw data or microarray SNP genotyping data as inputs, and reports to the users how the variants in their personal genomes impact the response to 193 drugs, including efficacy, dosage and toxicity.
