# Supplementary material for: Virtual Pharmacist: A Platform for Pharmacogenomics
Source: PLoS One. 2015 Oct 23;10(10):e0141105. doi: 10.1371/journal.pone.0141105 (PMC4619711; doi:10.1371/journal.pone.0141105)
Supplement: S2 File — It includes the detailed description of method for high-throughput data analysis and the strategy for handling SNPs in overlapping genes. (DOCX) [file pone.0141105.s002.docx]

**SNPs in overlapping genes**

There are occasions where one SNP affects two genes. We developed a pipeline to handle the SNPs located in the overlapping region of two genes (**REF**). Four of 324 SNPs need such handling. In our program, we report the drug response information for both overlapping genes affected by the same SNP.

Detail Strategy for handling overlapping genes

All of the drug response associated genes in our database (n = 190) were uploaded to the USCS genome browser to get the coordinates of each gene. We retrieved the overlapping regions according to the coordinates of each gene automatically by a Perl script. Five pairs of genes were identified to have overlapping regions. They are CLCN6 and MTHFR, CHRNA3 and CHRNA5, ERCC1 and CD3EAP, UGT1A1 and UGT1A6, ARVCF and COMT. For each pair of genes we retrieved the SNPs located on these genes from our database. Then, we checked whether the SNPs are located in the overlapping regions. For SNPs located at the overlapping regions, they were stored in the database as separate entries.


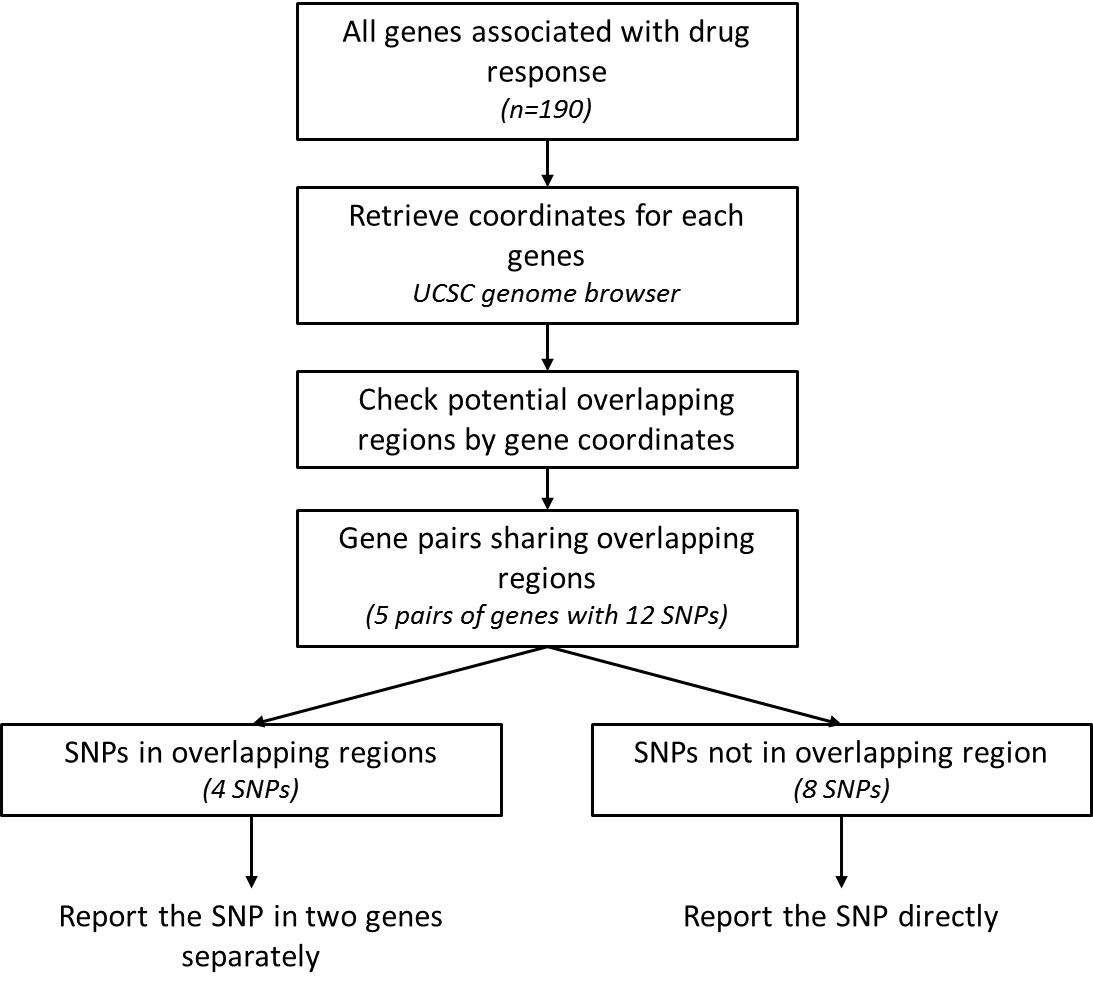


Fig. 1. Workflow for handling overlapping genes

Table 1. The detailed information of SNPs in overlapping genes

| Overlapping gene pair | overlapping region | Gene | Gene coordinate | drug response associated SNP IDs | SNPs lie in overlapping region? |
| --- | --- | --- | --- | --- | --- |
| CLCN6 and MTHFR | chr1:11866152-11866160 | CLCN6 | chr1:11866152-11903201 | rs1801133 | YES |
|  |  | MTHFR | chr1:11845786-11866160 | rs1801133 | YES |
|  |  |  |  | rs1801131 | NO |
|  |  |  |  | rs4846051 | NO |
| CHRNA3 and CHRNA5 | chr15:78885394-78887611 | CHRNA3 | chr15:78885394-78913637 | rs16969968 | YES |
|  |  | CHRNA5 | chr15:78857861-78887611 | rs16969968 | YES |
| ERCC1 and CD3EAP | chr19:45910590-45914024 | ERCC1 | chr19:45910590-45927177 | rs11615 | NO |
|  |  |  |  | rs3212986 | YES |
|  |  | CD3EAP | chr19:45909466-45914024 | rs3212986 | YES |
| UGT1A1 and UGT1A6 | chr2:234668918-234681945 | UGT1A1 | chr2:234668918-234681945 | rs4124874 | NO |
|  |  | UGT1A6 | chr2:234600320-234681945 | rs1105879 | NO |
| ARVCF and COMT | chr22:19957401-19957498 | ARVCF | chr22:19957401-20004309 | rs165599 | YES |
|  |  | COMT | chr22:19929262-19957498 | rs165599 | YES |
|  |  |  |  | rs9332377 | NO |
|  |  |  |  | rs4680 | NO |
|  |  |  |  | rs4646316 | NO |

**Collection of literatures studying cisplatin associated SNPs in different ethnicities**

We have carefully searched the literature and find 8 papers studying the cisplatin drug response in Asian, African and Caucasian population. The Asian and Caucasian population display similar trend in the drug response with 4 SNPs listed in the table below. There is only one study in African population.

Table 2. The detailed information of SNPs in overlapping genes

| RSID | GWAS reference in Asian population | GWAS reference in African population | GWAS reference in Caucasian population |
| --- | --- | --- | --- |
| rs1042522 | Genotypes CG + GG are associated with decreased response to cisplatin and paclitaxel in people with Stomach Neoplasms as compared to genotype CC.(PMID:19052714) |  | Genotype GG is associated with increased severity of Neutropenia when treated with cisplatin and cyclophosphamide in people with Ovarian Neoplasms as compared to genotypes CC + CG.(PMID:19786980) |
| rs11615 | Genotype GG is associated with increased overall survival when treated with fluorouracil, leucovorin and oxaliplatin in people with Colorectal Neoplasms as compared to genotypes AA + AG.(PMID:21057378) | Genotype AA is associated with increased risk of dying when treated with Platinum compounds in people with Colorectal Neoplasms as compared to genotype GG.(PMID:15213713) | Genotype GG is associated with decreased likelihood of nephrotoxicity when treated with cisplatin in people with Neoplasms as compared to genotypes AA + AG.(PMID:21902499) |
| rs316019 | Genotype CC is associated with increased clearance of metformin in healthy individuals as compared to genotype AC.(PMID:23417334) |  | Genotype CA is associated with decreased risk of nephrotoxicity when treated with cisplatin in people with neoplasms as compared to genotype CC.(PMID:19625999) |
| rs3957357 | Genotypes AA + AG are associated with increased likelihood of Anemia when treated with cisplatin and cyclophosphamide in women with Ovarian Neoplasms as compared to genotype GG.(PMID:22188361) |  | Genotypes AA + AG are associated with increased likelihood of Anemia when treated with cisplatin and cyclophosphamide in women with Ovarian Neoplasms as compared to genotype GG.(PMID:22188361) |

**Command lines for processing high throughput sequencing data**

#################################################################################

#

#the file describes all of command lines used for high-throughput sequence data

#

#input: DNA sequencing data (fastq format)

#

#output: Variatn call format (filtered VCF file)

#

#

#software prerequisite:

#1: BWA (Burrows-Wheeler Aligner) must be downloaded and installed. Package download from (http://bio-bwa.sourceforge.net/)

#2: picared tools: VP has implemented all binary files of picard tools (store in ./bin/picard-tools-1.119/). You can also download them from http://sourceforge.net/projects/picard/files/picard-tools/

#3: GenomeAnalysisToolKit: VP has implemented binary file of GATK. (store in ./bin/GenomeAnalysisTK.jar). You can also download them from https://www.broadinstitute.org/gatk/

#

#preparing for the reference data:

#1:reference genome:

#1): VP uses human_g1k_v37.fasta as reference genome.

# The reference genome must be indexed properly so as to perform the following analysis

# bwa index -a bwtsw human_g1k_v37.fasta (prepare for BWA alingment)

# samtools faidx human_g1k_v37.fasta (prepare for BWA alingment)

# java -Xmx2g -jar /path_to_picard/CreateSequenceDictionary.jar R=human_g1k_v37.fasta O=human_g1k_v37.dict (prepare for GATK SNP calling)

#

data_dir=$1 #path to VP package

data_name=$2 #name of the input data file in .fq format

account=$3 #user account of VP. By default. the uploaded sequencing data would be stored in ./ftp/your_user_account/

#BWA alignment: -t indicates the number of processors used for alignment. You can adjust it according to your server configuration

bwa aln $data_dir/bin/hg19/human_g1k_v37.fasta $data_dir/ftp/$account/$data_name -t 20 -I > $data_dir/ftp/$account/$data_name.sai

#generate sam file

bwa samse $data_dir/bin/hg19/human_g1k_v37.fasta $data_dir/ftp/$account/$data_name.sai $data_dir/ftp/$account/$data_name > $data_dir/ftp/$account/$data_name.sam

#reorder the sam file

java -Xmx50g -jar $data_dir/bin/picard-tools-1.119/ReorderSam.jar I=$data_dir/ftp/$account/$data_name.sam O=$data_dir/ftp/$account/$data_name.reorder.sam REFERENCE=$data_dir/bin/hg19/human_g1k_v37.fasta

#generate bam file

samtools view -bS $data_dir/ftp/$account/$data_name.reorder.sam > $data_dir/ftp/$account/$data_name.reorder.bam

#sort bam file

samtools sort $data_dir/ftp/$account/$data_name.reorder.bam $data_dir/ftp/$account/$data_name.reorder.sort

#add a header section of bam file so as to be recognized by GATK properly. remarkably the exact contend of header section has nothing to do with SNP calling by GATK

java -Xmx50g -jar $data_dir/bin/picard-tools-1.119/AddOrReplaceReadGroups.jar \

I=$data_dir/ftp/$account/$data_name.reorder.sort.bam O=$data_dir/ftp/$account/$data_name.reorder.sort.header.bam \

ID=hg19ID LB=hg19ID PL=illumina PU=hg19PU SM=hg19

#mark duplication

java -Xmx50g -jar $data_dir/bin/picard-tools-1.119/MarkDuplicates.jar REMOVE_DUPLICATES=true \

MAX_FILE_HANDLES_FOR_READ_ENDS_MAP=8000 INPUT=$data_dir/ftp/$account/$data_name.reorder.sort.header.bam OUTPUT=$data_dir/ftp/$account/$data_name.reorder.sort.header.dedup.bam \

METRICS_FILE=$data_dir/ftp/$account/$data_name.reorder.sort.header.dedup.metrics

#index the bam file

samtools index $data_dir/ftp/$account/$data_name.reorder.sort.header.dedup.bam

######################################################################

#base quality scre recalibration.

#

#three datasets are regarded as the training set for recalibration.

#

#it can be downloaed from GATK ftp site: ftp://ftp.broadinstitute.org/bundle/2.8/b37/

#

java -Xmx50g -jar $data_dir/bin/GenomeAnalysisTK.jar -T BaseRecalibrator -R $data_dir/bin/hg19/human_g1k_v37.fasta \

-I $data_dir/ftp/$account/$data_name.reorder.sort.header.dedup.bam -knownSites $data_dir/bin/hg19/dbsnp_138.b37.vcf \

-knownSites $data_dir/bin/hg19/Mills_and_1000G_gold_standard.indels.b37.vcf \

-knownSites $data_dir/bin/hg19/1000G_omni2.5.b37.vcf \

-o $data_dir/ftp/$account/$data_name.reorder.sort.dedup.bam.grp

java -Xmx50g -jar $data_dir/bin/GenomeAnalysisTK.jar -T BaseRecalibrator -R $data_dir/bin/hg19/human_g1k_v37.fasta \

-I $data_dir/ftp/$account/$data_name.reorder.sort.header.dedup.bam -BQSR $data_dir/ftp/$account/$data_name.reorder.sort.dedup.bam.grp \

-o $data_dir/ftp/$account/$data_name.reorder.sort.dedup.bam.grp1 \

-knownSites $data_dir/bin/hg19/dbsnp_138.b37.vcf \

-knownSites $data_dir/bin/hg19/Mills_and_1000G_gold_standard.indels.b37.vcf \

-knownSites $data_dir/bin/hg19/1000G_omni2.5.b37.vcf

java -Xmx50g -jar $data_dir/bin/GenomeAnalysisTK.jar -T PrintReads -R $data_dir/bin/hg19/human_g1k_v37.fasta \

-I $data_dir/ftp/$account/$data_name.reorder.sort.header.dedup.bam -BQSR $data_dir/ftp/$account/$data_name.reorder.sort.dedup.bam.grp \

-o $data_dir/ftp/$account/$data_name.reorder.sort.dedup.bam.grp.bam

#

java -Xmx50g -jar $data_dir/bin/GenomeAnalysisTK.jar -T AnalyzeCovariates -R $data_dir/bin/hg19/human_g1k_v37.fasta \

-before $data_dir/ftp/$account/$data_name.reorder.sort.dedup.bam.grp \

-after $data_dir/ftp/$account/$data_name.reorder.sort.dedup.bam.grp1 \

-csv $data_dir/ftp/$account/$data_name.reorder.sort.dedup.bam.grp.csv \

-plots $data_dir/ftp/$account/$data_name.reorder.sort.dedup.bam.grp.pdf

#

#finish of base quality score recalibration

#################################################################3

#############################################################

# SNP calling (unfiltered vcf) UnifiedGenotyper

#

#-stand_call_conf defined as the minimum confidence threshold (phred-scaled) at which the program should emit sites that appear to be possibly variant.

#-stand_emit_conf defined as the minimum confidence threshold (phred-scaled) at which the program should emit variant sites as called.

java -Xmx50g -jar $data_dir/bin/GenomeAnalysisTK.jar -T UnifiedGenotyper -R $data_dir/bin/hg19/human_g1k_v37.fasta \

-I $data_dir/ftp/$account/$data_name.reorder.sort.dedup.bam.grp.bam \

-stand_call_conf 30 -stand_emit_conf 10 \

-metrics $data_dir/ftp/$account/$data_name.reorder.sort.header.dedup.metrics \

-o $data_dir/ftp/$account/$data_name.raw_variants.vcf

#select the SNPs from the original raw variant file

#because all of the variants associated with drug response in our database are SNPs, so we extract them seperately so as to increase the processing speed

java -jar $data_dir/bin/GenomeAnalysisTK.jar -T SelectVariants -R $data_dir/bin/hg19/human_g1k_v37.fasta \

-V $data_dir/ftp/$account/$data_name.raw_variants.vcf -selectType SNP \

-o $data_dir/ftp/$account/$data_name.raw_snps.vcf

#

#SNPs filtering

#QualByDepth(QD): This is the variant confidence (from the QUAL field) divided by the unfiltered depth of non-reference samples.

#RMSMappingQuality(MQ): This is the Root Mean Square of the mapping quality of the reads across all samples.

java -jar $data_dir/bin/GenomeAnalysisTK.jar -T VariantFiltration -R $data_dir/bin/hg19/human_g1k_v37.fasta \

-V $data_dir/ftp/$account/$data_name.raw_snps.vcf \

--filterExpression "QD < 2.0 || MQ < 60.0 " \

--filterName "my_snp_filter" \

-o $data_dir/ftp/$account/$data_name.filtered_snps.vcf
